# TAp73β Can Promote Hepatocellular Carcinoma Dedifferentiation

**DOI:** 10.3390/cancers13040783

**Published:** 2021-02-13

**Authors:** Evin Iscan, Umut Ekin, Gokhan Yildiz, Ozden Oz, Umur Keles, Aslı Suner, Gulcin Cakan-Akdogan, Gunes Ozhan, Marta Nekulova, Borivoj Vojtesek, Hamdiye Uzuner, Gökhan Karakülah, Hani Alotaibi, Mehmet Ozturk

**Affiliations:** 1 Izmir Biomedicine and Genome Center, Izmir 35000, Turkey; evin.iscan@ibg.edu.tr (E.I.); umut.ekin@ibg.edu.tr (U.E.); ozden.oz@ibg.edu.tr (O.O.); umur.keles@ibg.edu.tr (U.K.); gulcin.cakan@ibg.edu.tr (G.C.-A.); gunes.ozhan@ibg.edu.tr (G.O.); hamdiye.uzuner@msfr.ibg.edu.tr (H.U.); gokhan.karakulah@ibg.edu.tr (G.K.); hani.alotaibi@ibg.edu.tr (H.A.); 2 Izmir International Biomedicine and Genome Institute, Dokuz Eylul University, Izmir 35000, Turkey; 3 Department of Medical Biology, Faculty of Medicine, Karadeniz Technical University, Trabzon 61000, Turkey; gokhanyildiz@ktu.edu.tr; 4 Izmir Bozyaka Education and Research Hospital, University of Health Sciences, Izmir 35000, Turkey; 5 Department of Biostatistics and Medical Informatics, Faculty of Medicine, Ege University, Izmir 35000, Turkey; asli.suner@ege.edu.tr; 6 Department of Medical Biology, Faculty of Medicine, Dokuz Eylul University, Izmir 35000, Turkey; 7 RECAMO, Masaryk Memorial Cancer Institute, 60200 Brno, Czech Republic; m.nekulova@seznam.cz (M.N.); vojtesek@mou.cz (B.V.)

**Keywords:** TAp73, hepatocellular carcinoma, metastasis, dedifferentiation, zebrafish, yes-associated protein 1

## Abstract

**Simple Summary:**

Hepatocellular carcinoma (HCC) is a highly complex and heterogeneous type of cancer. Hepatocyte dedifferentiation is one of the important steps in the development of HCC. However, its molecular mechanisms are not well known. In this study, we report that transcriptionally active TAp73 isoforms are overexpressed in HCC. We also show that TAp73β suppresses the expression of the hepatocyte markers including CYP3A4, AFP, ALB, HNF4α, while increasing the expression of several cholangiocyte markers in HCC cell lines. In conclusion, this report reveals a pro-oncogenic role for TAp73β in liver cancer.

**Abstract:**

Hepatocyte dedifferentiation is a major source of hepatocellular carcinoma (HCC), but its mechanisms are unknown. We explored the p73 expression in HCC tumors and studied the effects of transcriptionally active p73β (TAp73β) in HCC cells. Expression profiles of p73 and patient clinical data were collected from the Genomic Data Commons (GDC) data portal and the TSVdb database, respectively. Global gene expression profiles were determined by pan-genomic 54K microarrays. The Gene Set Enrichment Analysis method was used to identify TAp73β-regulated gene sets. The effects of TAp73 isoforms were analyzed in monolayer cell culture, 3D-cell culture and xenograft models in zebrafish using western blot, flow cytometry, fluorescence imaging, real-time polymerase chain reaction (RT-PCR), immunohistochemistry and morphological examination. TAp73 isoforms were significantly upregulated in HCC, and high p73 expression correlated with poor patient survival. The induced expression of TAp73β caused landscape expression changes in genes involved in growth signaling, cell cycle, stress response, immunity, metabolism and development. Hep3B cells overexpressing TAp73β had lost hepatocyte lineage biomarkers including ALB, CYP3A4, AFP, HNF4α. In contrast, TAp73β upregulated genes promoting cholangiocyte lineage such as YAP, JAG1 and ZO-1, accompanied with an increase in metastatic ability. Our findings suggest that TAp73β may promote malignant dedifferentiation of HCC cells.

## 1. Introduction

The primary liver cancers that comprise hepatocellular carcinoma (HCC), intrahepatic and mixed cholangiocarcinomas, fibrolamellar HCC and hepatoblastoma display high cellular heterogeneity, including stem-like, cholangiocyte-like and hepatocyte-like cells [1,2]. Hepatic stem/progenitor cells are bi-potential, thus able to generate both hepatocytes and cholangiocytes [3]. Accordingly, primary liver cancers might originate from such progenitor cells [4], but they also originate from hepatocytes [1,5,6,7]. According to hepatocyte origin hypothesis, originator hepatocytes need to undergo dedifferentiation and/or transdifferentiation to create the cellular heterogeneity observed in primary liver cancers. The mechanisms driving such dedifferentiation or transdifferentiation processes are largely unknown.

The p73 gene is capable of encoding a large set of transcript isoforms, allowing the synthesis of a central DNA binding domain, which is associated with modified N-terminal and C-terminal regions [8,9]. The implications of C-terminal modifications are not well understood, while N-terminally modified p73 isoforms display opposite functions [10,11,12,13,14]. N-terminally modified isoforms differ from each other principally by the presence or absence of a transactivation (TA) domain [8,14,15,16]. The so-called TAp73 isoforms act as activators of transcription; the DNp73 isoforms lacking the TA domain cannot activate transcription. There are also reports that DNp73 isoforms display a dominant negative effect over TAp73 isoforms [10,11,12].

As a close relative of the tumor suppressor gene *p53*, *p73* has been extensively explored for its implications in cancer [17,18,19,20]. As opposed to *p53*, the *p73* gene is not mutated in cancer, but its expression might be affected [14,17,19]. TAp73 isoforms, being able to induce apoptosis or cell cycle inhibition experimentally, are considered to be tumor suppressor, whereas different DNp73 isoforms have often been qualified as “oncogenic” [8,10,13,14,21]. Many studies have reported an upregulation of DNp73 isoforms in cancer as a mechanism to counteract tumor suppressor functions of TAp73 isoforms [9,10,11,12]. In addition, DNp73 isoforms are considered as markers of poor prognosis [22,23,24,25]. The status of TAp73 isoforms in tumors is not clear and their potential roles in cancer malignancy are poorly understood.

Our goal was to reexamine the role of p73 in HCC. Using a large set of HCC tumors from the GDC data portal, we demonstrate that TAp73 isoforms are strongly induced in HCC, and p73 overexpression is associated with poor survival. These unexpected findings led us to re-examine the role of transcriptionally active p73 in HCC. We demonstrate, for the first time, that TAp73β isoform promotes dedifferentiation of hepatocyte-like cells by repressing the expression of hepatocyte-specific biomarkers and by activating genes involved in cholangiocyte lineage development.

## 2. Results

### 2.1. TAp73 Is Upregulated in HCC and Associated with Poor Patient Survival

**Figure 1:**
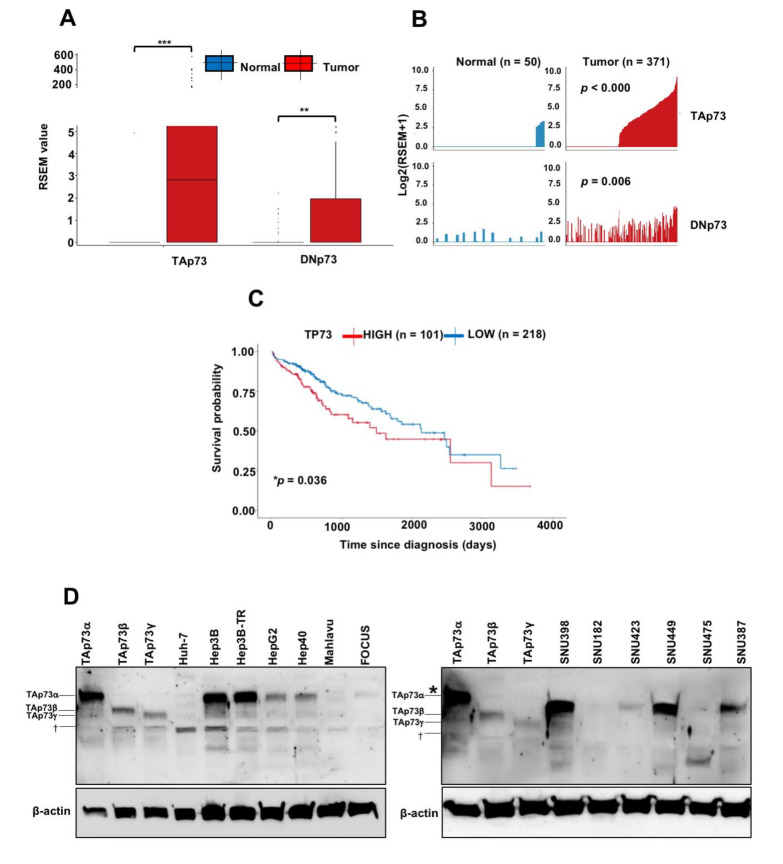
Strongly induced expression of TAp73 isoforms in hepatocellular carcinoma and poor survival of patients with a high p73 expression. (**A**) Box plots of p73 isoforms (TAp73 *versus* DNp73 isoforms) expression in non-tumor liver (normal; n = 50) and hepatocellular carcinoma (tumor; n = 371) were collected from the TSVdb database. *** *p* < 0.001 and ** *p* = 0.006 for significant differences between normal and tumor samples. (**B**) Bar plots of TAp73 and DNp73 isoforms distribution in normal and tumor samples. TAp73: Tumor %53.1 (n = 197/371)—Normal %8.0 (n = 4/50); DNp73: Tumor %37.4 (n = 139/371)—Normal %24.0 (n = 12/50) (**C**) Survival curves of liver cancer patients as a p73 high expression (n = 101), versus p73 low expression (n = 218). * *p* < 0.05. (**D**) p73 isoform expression in HCC cell lines was examined by western blot using a primary antibody against the full-length p73, which detects both TAp73 and DNp73 isoforms [27] and β-actin was used as a loading control (n = 2). p73 isoforms endogenously expressed in HCC cell lines were identified by comparing their relative migrations in the SDS-PAGE system in comparison with ectopically expressed TAp73α, TAp73β and TAp73γ isoforms in Huh-7 cells using mammalian expression vectors. † Non-specific signals. * The apperant shift observed here is due to a problem of gel migration.

In order to explore the isoform level expression of p73 in a normal liver and HCC, we used the TCGA Splice Variant database (TSVdb); a web-tool for the differential analysis of p73 transcript isoforms in HCC as compared to non-tumor liver tissues in TCGA data [26]. The total sample size comprised 371 tumors and 50 non-tumor tissues. The web-tool allowed us to simultaneously analyze the expression of TAp73 and DNp73 isoforms respectively. The IBM SPSS Version 25.0 statistical package program (IBM Corporation, Armonk, NY, USA) was used for the determination of median, interquartile range, mean rank and *p*-values. Based on median values, there was no significant expression of any p73 isoform in the liver. In contrast, the TAp73 isoforms expression were increased in HCC tumors highly significantly (*p* = 2.9^−9^). DNp73 isoforms were also upregulated, but less significantly (*p* = 0.006315) as shown in Figure 1A. Relative expressions of TAp73 and DNp73 isoforms showed moderate correlation (*r =* 0.7 *),* as shown in Figure 1B. Next, we examined patient survival according to total p73 expression, as no data was available for isoform-specific expression. HCC patients with a high p73 expression displayed poor survival (*p* = 0.036) as shown in Figure 1C.

To confirm the expression of p73 isoforms at the protein levels in HCC cells, we used a primary antibody against the full-length p73, which detects both TAp73 and DNp73 isoforms [27]. As shown in Figure 1D, eight HCC cell lines expressed TAp73α, at high levels (Hep3B, Hep3B-TR, SNU398, SNU449, SNU387) or moderately (HepG2, Hep40, SNU423). TAp73 isoforms expression was weakly detectable or undetectable in five other cell lines (Huh-7, FOCUS, Mahlavu, SNU182, SNU475), TAp73β and TAp73γ isoforms were detected only in SNU398 cells which also displayed the highest expression of TAp73α. We could not detect any DNp73 isoform expression in the same set of HCC cell lines. Taken together, these observations indicate acquired TAp73 expression in HCC cells.

### 2.2. TAp73β Induction Leads to Profound Changes in Global Gene Expression Affects Multiple Gene Sets and Cellular Processes in HCC Cells

**Figure 2:**
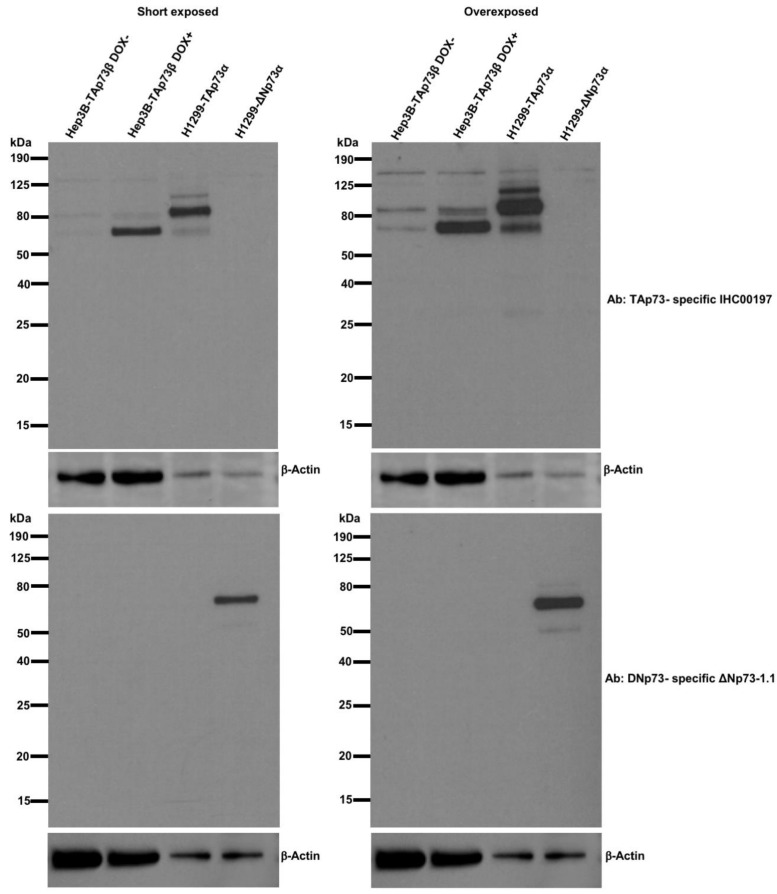
T-Rex-inducible expression in Hep3B cells resulted in TAp73β overexpression, with no detectable expression of DNp73 protein. Hep3B cells with (Hep3BTAp73β DOX+) or without (Hep3BTAp73β DOX−) doxycycline treatment were compared to H1299 transiently transfected with TAp73α (H1299-TAp73α) or DNp73 (H1299-DNp73). Western blot analysis of cell lysates using IHC00197 antibody recognizing TAp73 isoforms (top panel) and western blot analysis of cell lysates using ΔNp73-1.1 antibody recognizing DNp73 isoforms [31,32] (bottom panel). β-actin antibody was using as a loading control.

Based on the overexpression of TAp73 isoforms in HCC tumors and some HCC cell lines, we decided to further investigate the role of transcriptionally active p73 in HCC by overexpressing TAp73β in Hep3B cells because they display a deletion of *TP53* gene resulting in the loss of expression of wild type p53 protein [28]. Huh7 cells were not preferred because they strongly express mutant Y220C p53 protein [15]. To minimize the effects of cellular context, we used a doxycycline-induced protein expression system. As described previously, wild type p53 and TAp73 share common targets [29] and mutant p53 forms may interfere with TAp73 cellular effects [30]. Thus, Hep3B cells were most appropriate model for us to study TAp73β effects in HCC. As shown Figure 2, Hep3B cells used for this construction in the laboratory of one of us (B.V.) expressed low level of TAp73 but not DNp73 endogenously (Figure 2; Hep3B-TAp73β DOX−). Doxycyline treatment of these cells resulted in the induction of TAp73 but not DNp73 isoform expression (Figure 2; Hep3B-TAp73β DOX+).

For gene expression analysis, the expression of TAp73β was induced in Hep3B cells by treating the cells with doxycycline up to 48 h. We collected total RNAs at 12 h, 24 h, and 48 h from induced and non-induced cells. We compared global gene expression profiles by gene expression analysis of these RNA samples with pan-genomic 54K Affymetrix microarrays. A total of 14 independent biological replicates were used. At 48 h, three replicates from each treatment group were analyzed; at earlier time points (12 h and 24 h, respectively), two replicates of each treatment were included. The combined analyses of 14 biological samples identified a total of 877 genes that showed two-fold or a higher expression difference in at least one time-point under ectopic TAp73β expression. Of these 877 genes, 578 (66%) were up-regulated and 299 (34%) were down-regulated (shown in Table S1). Next, we calculated the Gene Set Enrichment Analysis (GSEA) enrichment scores for control and p73-induced samples at 48 h using different gene set collections available at the Molecular Signatures Database (MSigDB) [33]. Based on significant nominal *p* values (<0.001) and the false discovery rate (FDR)FDR (<0.25), enrichments were observed with many gene set collections (shown in Table S1). Here, we focused on data obtained with the “Hallmark Gene Sets” collection, where 30 hallmark gene sets were enriched (shown in Table S2). TAp73β -affected hallmark gene sets clustered into six distinct processes, namely stress response, growth signaling, cell cycle, development, immune response and metabolism (shown in Table S3).

### 2.3. Inducible TAp73β Overexpression Inhibits Cell Proliferation, but Does Not Induce Cell Death

**Figure 3:**
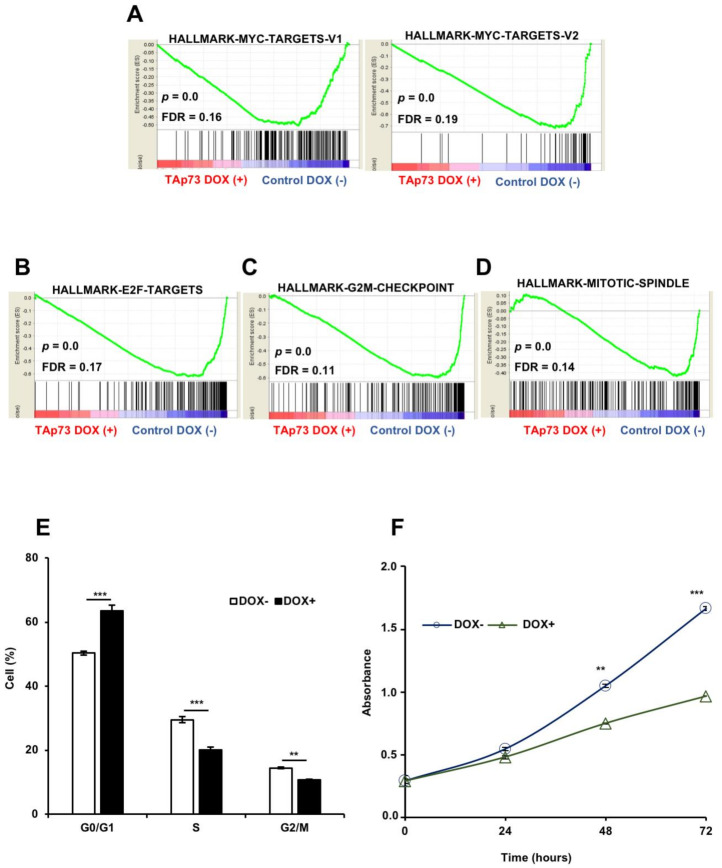
TAp73β down-regulates cell proliferation by increasing the proportion of cells at G0/G1 phase at the expense of S and G2/M phase cells. Gene set enrichment analyses showing the repression of (**A**) Myc-target genes, (**B**) E2F target genes, (**C**) genes involved in G2/M cell cycle progression, and (**D**) mitotic spindle associated genes. (**E**) TAp73β causes moderate inhibition of cell cyle progression by increasing the relative number of cells at G0/G1 phase. Hep3BTAp73β cells were treated with doxycycline (1 µg/mL) for 72 h, and cell cycle distribution was evaluated by using flow cytometric DNA content analysis. (**F**) TAp73β decreases cell proliferation by 40%, but does not stop cell growth. Hep3BTAp73β cells were exposed to doxycycline (1 µg/mL) for 0 h, 24 h, 48 h and 72 h. At the end of the indicated times, cell proliferation was measured by the SRB method. Columns and error bars indicate ± SD from triplicate experiments. ** *p* < 0.001 and *** *p* < 0.0001 for significant differences between cells treated and not treated with doxycycline.

As expected, genes involved in the p53 pathway and related networks were induced, whereas genes regulated by MYC were repressed by TAp73β. Cell cycle regulatory genes such as E2F transcription factor targets and genes involved in G2/M checkpoint and mitotic spindle assembly were likewise repressed (shown in Figure 3A–D). In accordance with these expression changes, we observed significant deviations in the cell cycle-stage distribution of cells, as manifested by an increase of G0/G1 phase cells at the expense of S and G2/M phase cells (shown in Figure 3E). We also noticed the lack of accumulation in sub G1 cells, suggesting the absence of significant apoptosis (shown in Figure S1A). We confirmed this finding by demonstrating that the TAp73β induction did not affect the Annexin V staining pattern at 72 h (shown in Figure S1B). Taken together, these results clearly indicated that TAp73β overexpression in HCC cells down-regulates cell proliferation by decreasing the relative number of cycling cells with no permanent cell cycle arrest, nor cell death. In confirmation, TAp73β induction decreased the proliferation rate of Hep3B cells (shown in Figure 3F) without affecting their survival for at least 5 days (see later Figure 6).

### 2.4. TAp73β Activates YAP, but Not Bax Expression

**Figure 4:**
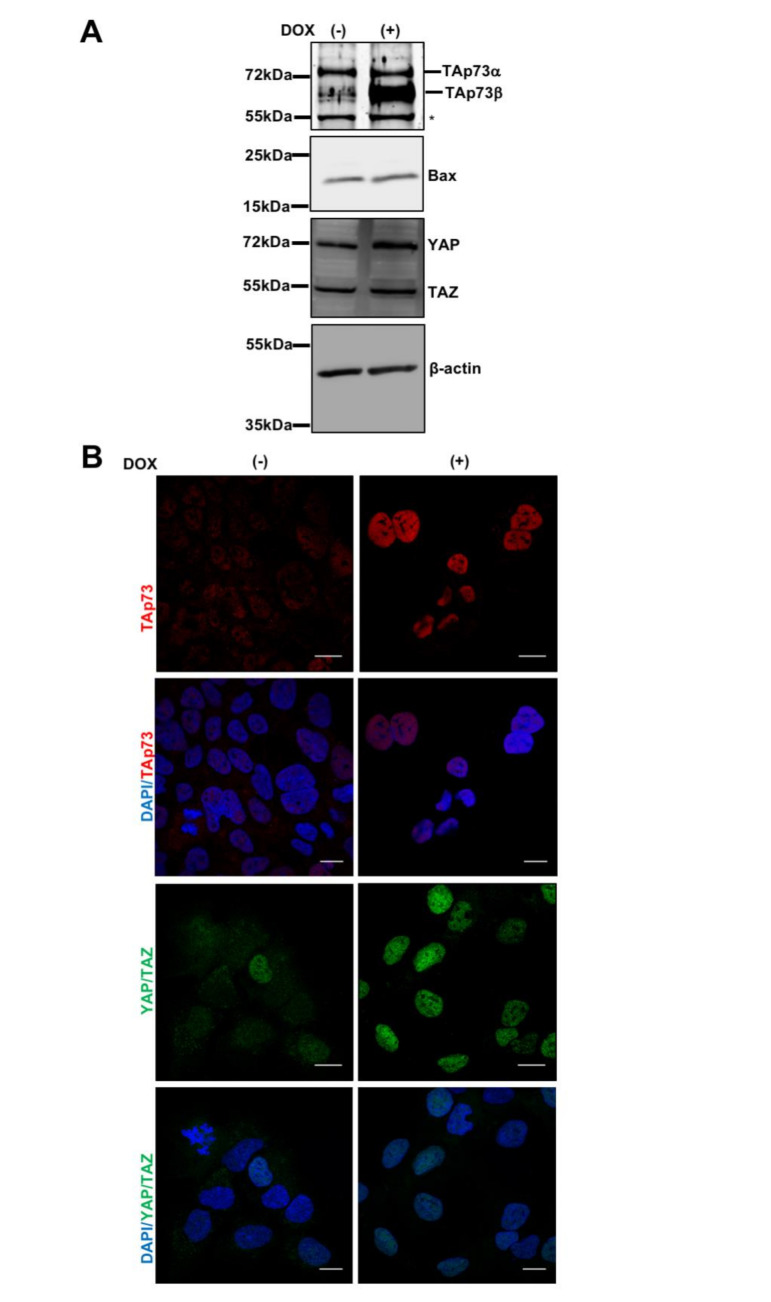
TAp73β activates expression and nuclear localization of YAP, but does not affect Bax expression. (**A**) Hep3BTAp73β cells were treated with doxycycline (1 µg/mL) for 72 h and cell lysates were obtained, then western blot was performed for p73, YAP and Bax expression. β-actin was used as a loading control (n = 2). * Non-specific signals. (**B**) Upon TAp73β induction, both TAp73 and YAP show nuclear accumulation (*). Immunofluorescence assay was performed to determine the expression and localization of p73 (Red), YAP (Green), respectively in Hep3BTAp73β cells treated with Doxycycline (1 µg/mL) for 72 h. DAPI (blue) was used for nuclear counterstain. Images were taken at x63 magnification in a confocal microscope. Scale bar = 20 μm. * Please note that the antibody used for TAp73β immunofluorescence recognizes all p73 isoforms [27]. Similarly, D24E4 antibody used for YAP immunofluorescence also detects TAZ protein.

Adenoviral transfer of TAp73β in Hep3B cells has been reported to Bax gene induction followed by apoptosis [11]. It has also been reported that induction of Bax expression is mediated by TAp73 association with YAP (Yes-associated protein 1) co-activator at the nuclei [34,35,36]. Therefore, we checked Bax and YAP expression in our experimental system, following TAp73β induction (Figure 4). Under our experimental conditions there was no Bax accumulation. In contrast, western blotting assay using D24E4 antibody recognizing both YAP and related TAZ (WW domain containing transcription regulator 1) showed a slight increase in total YAP protein levels but not TAZ protein (shown in Figure 4A). Activated YAP migrates into nucleus and acts as transcriptional regulator. Accordingly, indirect immunofluorescence assay showed that both YAP and TAp73β accumulate in nuclei of Hep3B cells following TAp73β induction (Figure 4B).

### 2.5. TAp73β Overexpression Causes Dedifferentiation of HCC Cells by Repressing Hepatocyte Lineage Markers and Inducing Cholangiocyte Lineage Promoters

**Figure 5:**
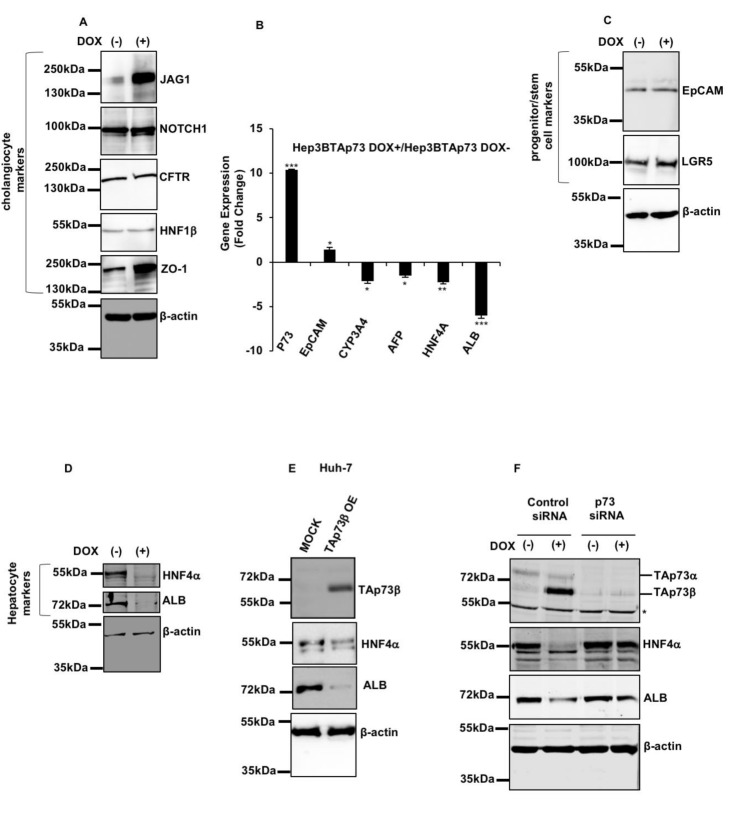
TAp73β induced the expression of cholangiocyte promoting genes (JAG1, NOTCH1, ZO-1) while repressing hepatocyte fate markers (CYP3A4, AFP, HNF4α, ALB) without affecting progenitor/stem marker (EpCAM, LGR5) expression. Hep3BTAp73β cells were treated with Doxycycline (1 µg/mL) to induce TAp73β expression for 72 h and cell lysates and RNA were obtained, then western blot and RT-PCR were performed. (**A**) Western blotting of cholangiocyte markers JAG1, NOTCH1, CFTR, HNF1β, ZO-1 (n = 2). (**B**) EpCAM progenitor/stem cell marker and hepatocyte markers, *CYP3A4*, *AFP*, *HNF4*α and *ALB* expressions were examined by RT-PCR. GAPDH was used as a housekeeping gene. Column graphs show the fold change and calculated by using the 2^−∆∆Ct^ method. * *p* < 0.05, ** *p* < 0.001 and *** *p* < 0.0001. (**C**) Western blotting of progenitor/stem markers EpCAM, and LGR5 (n = 2). (**D**) Western blotting of hepatocyte markers ALB and HNF4α (n = 2). (**E**) Huh7 cells were transiently transfected with TAp73β and, HNF4α and ALB protein levels were studied by western blotting. (**F**) Doxycyline-treated and untreated Hep3BTAp73β cells were transfected with p73siRNA and scrambled siRNA, respectively. Western blot analysis for p73, HNF4α and ALB expression in control siRNA and p73 siRNA transfected Hep3BTAp73β cells with or without doxycycline (1 µg/mL) induction (n = 2). * Non-specific signals.

**Figure 6:**
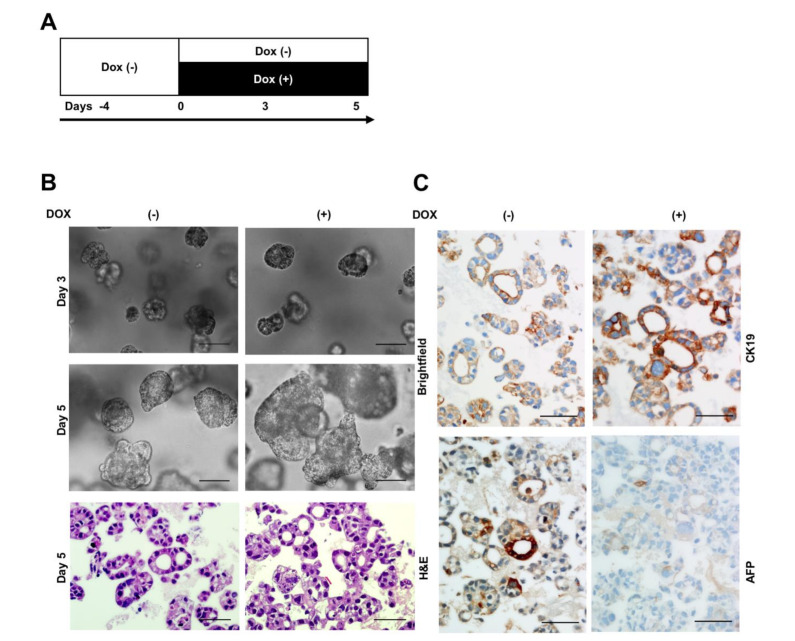
TAp73β represses AFP^+^ hepatocyte-like 3D cell structures formation while stimulating CK19^+^ cholangiocyte-like 3D cell structures formation. (**A**) A scheme depicting Hep3BTAp73β cells grown in the absence of doxycycline (DOX−) in Matrigel for 4 days, followed by additional growth in the absence (Dox−) or presence (DOX+) of doxycycline (1 µg/mL) for 5 days. (**B**) Brightfield images of differentially treated 3D cell structures at days 3 and 5, and H&E staining at day 5. (**C**) Immunohistochemistry staining for CK19 and AFP which was performed using formaldehyde-fixed paraffin-embedded 3D cell structures that have been grown 5 days in the presence (DOX+) or absence (DOX−) of doxycycline. Images were taken at ×20 magnification in a brightfield microscope. Scale bar = 100 μm.

Based on the induction of YAP by TAp73β in Hep3B cells, we thoroughly examined the list of genes upregulated by TAp73β (Table S1), and identified several targets of YAP, including NOTCH1 and its ligand JAG1 that are involved in stemness and bile duct development [37,38,39,40]. Western blot analysis confirmed TAp73 induced expression of Jagged1 (JAG1) at protein level. However, TAp73β induction did not affect the NOTCH1 protein levels (both shown in Figure 5A). Next, we analyzed the expression of genes involved in hepatobiliary differentiation by the RT-PCR and western blot assays respectively. Hep3B cells expressed the stem cell and cholangiocyte-lineage markers zonula occludens-1 (ZO-1), epithelial cell adhesion molecule (EpCAM), leucine-rich repeat containing G protein-coupled receptor 5 (LGR5), cytokeratin 19 (CK19), hepatocyte nuclear factor-1 beta (HNF-1β), cystic fibrosis transmembrane conductance regulator (CFTR), as well as the hepatocyte lineage markers cytochrome P450 family 3 subfamily A member 4 (CYP3A4), albumin (ALB), hepatocyte nuclear factor 4 alpha (HNF4α) and alpha fetoprotein (shown in Figure 5A–D; for CK19 and AFP, see Figure 6 for immunocytochemical data). Upon TAp73β induction, the expression of some of these markers was profoundly affected. Notably, we observed a sharp increase in ZO-1, together with a decrease in *CYP34A*, *ALB, HNF4α,* and *AFP* transcript levels (shown in Figure 5A,B,D). There was no major change in some cholangiocyte markers including CFTR and HNF1β (shown in Figure 5A), and also in hepatic stem/progenitor markers including EpCAM and LGR5 at the protein levels (shown in Figure 5C).

Hep3B and Huh7 cells are composed of stem/progenitor cells [41,42] able to undergo partial differentiation to hepatocytes and cholangiocytes. Based on suppression of hepatocyte markers in Hep3B cells after TAp73β induction (Figure 5B,D), we also tested TAp73β effects in Huh7 cells by transient transfection assay and confirmed the suppression of hepatocyte marker expression (Figure 5E). The specificity of TAp73β effect on hepatocyte marker suppression was demonstrated by p73-specific siRNA, as shown in Figure 5F. Stimulated by exciting findings, we strived to further characterize the role of TAp73 overexpression in hepatobiliary fate change. We first optimized the growth of Hep3B cells as 3D cell culture by cultivating them in Matrigel for 9 days (shown in Figure 6A,B). Then we studied TAp73β-induced changes using a protocol shown in Figure 6A. Cells were left 4 days in standard culture medium, followed by 5 days of culture with or without Doxycycline. The phenotypic comparison of TAp73β -induced 3D cell structures with controls under brightfield microscopy and by hematoxylin and eosin (H&E) staining showed no major difference in their gross morphology. They formed mixed 3D cell structures with or without a lumen (shown in Figure 6B). We analyzed these 3D cell structures for the expression of AFP and CK19 as hepatocyte lineage and cholangiocyte lineage markers, respectively. In the absence of TAp73β overexpression, both markers displayed a heterogeneous expression (shown in Figure 6C-left panel). When exposed to TAp73β overexpression for 5 days, many 3D cell structures with a lumen displayed strong basolateral CK19 staining. Under the same conditions, 3D cell structures became fully negative for AFP immunostaining (shown in Figure 6C-right panel). Among other markers tested, β-catenin was strongly and homogeneously positive for all 3D cell structures. Rare CK18-positive Hep3B 3D cell structures became less abundant under TAp73β induction. A few arginase-positive 3D cell structures were not affected. Finally, there was 3D cell structures positive for mature hepatocyte marker HepPAR-1, independent from TAp73β expression (shown in Figure S2).

### 2.6. TAp73β Overexpression Promotes HCC Metastasis

**Figure 7:**
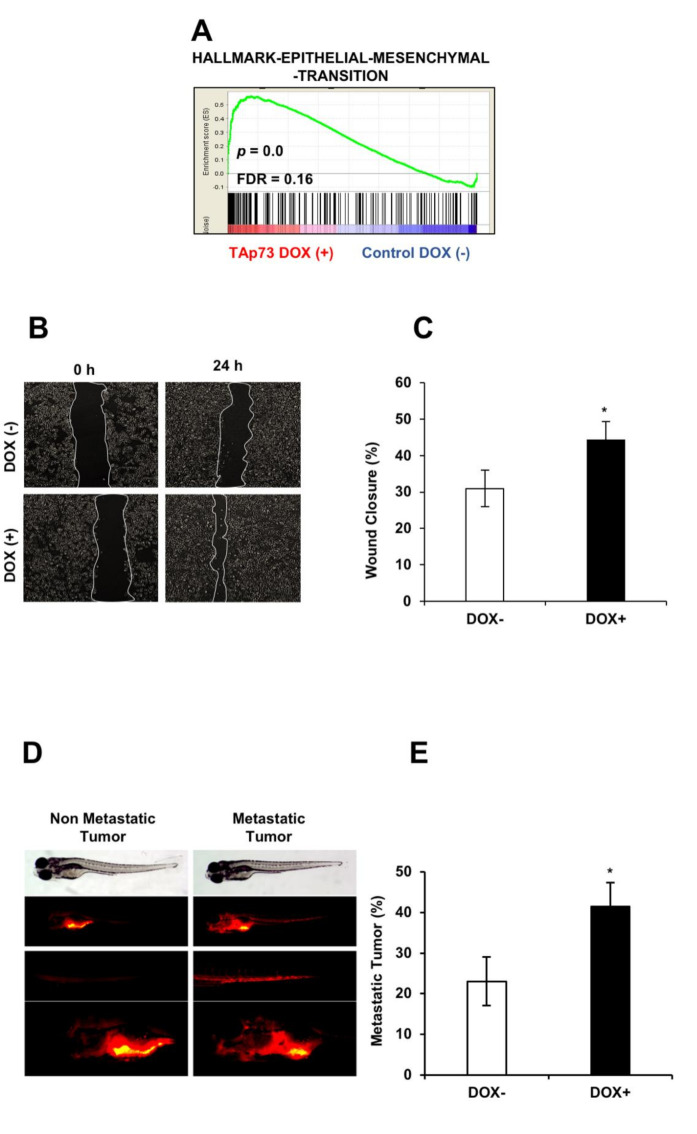
TAp73β upregulates epithelial-mesenchymal transition hallmark genes and stimulates metastatic abilities of hepatocellular carcinoma cells. (**A**) Genes involved in epithelial-mesenchymal transition in TAp73β induced cells. (**B**) Wound-healing assay was performed to examine the migration rate of Hep3B cells under TAp73β induction. Images were captured at 0 and 24 h following the initial scratch. (**C**) Migration rates were quantified by measuring three different wound areas using ImageJ software, columns and error bars indicate mean ± SD from triplicate experiments. The graph is representative of three independent experiments. (**D**) This figure illustrates metastatic (right panel) and non-metastatic (left panel) tumors after 4 days grown in zebrafish larvae. Bright field picture of larvae was shown in the top level the bottom level pictures are fluorescent images of tumors in same animals. Fluorescent pictures in the middle shows metastatic tumor cells disseminated into tail region (right) as opposed to non-metastatic tumor cells (left). Hep3BTAp73β cells were treated with doxycycline 1 µg/mL for 72 h, stained with Dil and inoculated into the 2 dpf zebrafish embryos. Bright-field and fluorescent images of zebrafish were captured for non-metastatic and metastatic tumors. The number of embryos with metastasis was counted at day 4 dpi (days post-injection) (**E**). Three separate experiments were performed. Error bars, ± SD (number of zebrafish embryo, n = 30). * *p* < 0.05.

Our GSEA analysis revealed that TAp73β induced the epithelial-to-mesenchymal transition (EMT) related hallmark gene sets (Figure 7A). Therefore, we investigated whether TAp73β −induced EMT contributes to the migration and metastasis of HCC cells. Firstly, we performed wound healing assays to examine the effect of TAp73β on cell migration. TAp73β increased cell migration from 31% to 44% in 24h (Figure 7B,C). To test the effects of TAp73β overexpression on metastasis in vivo, we performed xenograft assay as reported previously [43]. To test the effects of TAp73β overexpression on metastasis in vivo, we performed xenograft assay as reported previously [43]. We found that control Hep3B cells metastasized to the tail and head in 21% of the zebrafish embryos tested. Induction of TAp73β expression caused a two-fold increase in their metastatic capacity, with tail and head invasion in 42% of the zebrafish embryos tested (shown in Figure 7D,E). Thus, TAp73 overexpression conferred a two-fold increase in metastatic capacity to HCC cells.

## 3. Discussion

Based on the analysis of a large dataset, we demonstrate that the p73 gene is weakly expressed in liver, while TAp73 isoforms are strongly and significantly upregulated in HCC tumors. More importantly, a high expression of p73 gene in HCC is a potential predictor of poor patient survival. Together, these findings imply a previously unsuspected promalignant role for TAp73 isoforms, which otherwise are considered as tumor suppressor. To test such a potentially promalignant role, we set up a series of experiments, using an experimental model based on HCC-derived Hep3B cells with doxycycline-regulated TAp73β expression. Over a period of 48 h, TAp73β was able to affect the expression of several hundred genes both positively and negatively. This change in global gene expression landscape pinpoints a multi-task role of TAp73β in HCC cells. Since the p53 gene is deleted [28], and the *p63* gene expression is very low [44] in these cells, we assume that TAp73β acted alone in this process. As demonstrated by GSEA, genes affected by TAp73β overexpression embraced a large spectrum of cellular events such as growth regulation, stress response, immune response, metabolism and development.

Under our experimental conditions, TAp73β overexpression resulted in YAP protein increase in cells, together with a nuclear accumulation of both TAp73β and YAP (more precisely YAP/TAZ since D24E4 recognized both proteins). However, these events did not lead to Bax induction, in accordance with the lack of an apoptotic response. This could be due to a cooperation between YAP and TEAD that is known to play anti-apoptotic role in cancer cells [45,46]. In the absence of drastic responses such as cell death or permanent growth arrest, we expanded our exploration to the additional effects of TAp73β. A group of TAp73 target gene sets are involved in development-related events. Therefore, we asked specifically whether TAp73β is able to provoke cell fate changes in HCC cells. The first clue came from the TAp73β-induced nuclear accumulation of YAP protein, which plays a crucial role in regulating tissue homeostasis and organ size [47]. Luckily, Hep3B cells harbor a subpopulation of stem/progenitor cells [41] as well as hepatocyte-like and cholangiocyte-like cells as revealed by the expression of EpCAM, LGR5, CYP3A4, AFP, ALB, HNF4α, HNF1β, NOTCH1, CK19, CFTR, ZO-1 (shown in Figure 5 and Figure 6). We established experimental proof for a robust effect of TAp73 on HCC cell fate by western blot and immunocytochemistry in Hep3B and Huh7 cells grown under 2D monolayer and 3D cell culture. Under TAp73-induced conditions, the expression of hepatocyte lineage markers was strongly suppressed and this suppression was partially reversed in p73-silenced cells (shown in Figure 5F). There was no major change in the expression of a stem cell marker, but several cholangiocyte markers including JAG1 and ZO-1 were also upregulated by TAp73β overexpression. When analyzed together, these findings indicated TAp73β overexpression represses hepatocyte differentiation, but stimulates cholangiocyte fate without affecting EpCAM^+^ and LGR5^+^ stem/progenitor cell population. This drastic effect of TAp73β results in a two-fold increase in the metastatic ability of HCC cells in the zebrafish larval xenograft model. These findings may also be relevant to poor patient survival associated with a high p73 expression in HCC tumors.

Some of our findings deserve further comment based on previously published reports. TAp73 and p53 share structural and functional similarities. The depletion of p53 in mature hepatocytes combined with YAP overexpression caused dedifferentiation to progenitor-like cells that gave rise to mixed HCC-CC tumors [5]. Our data indicate that the overexpression, rather deletion of TAp73β in human HCC cells inhibited hepatocyte lineage development, in favor of a cholangiocyte-like phenotype. As Hep3B cells deleted p53 and overexpress YAP, the effect we observed with TAp73β does not necessarily contradict the observations with p53 depletion [5]. The levels of TAp73β in p53-deleted mouse liver have not been reported, but p53 depletion in human cancer cells results in an upregulation of TAp73β levels [48]. Recent molecular studies provide evidence for a non-exclusive mechanism for the cell origin of HCC, implicating either dedifferentiation of mature hepatocytes or as an aberration of liver stem/progenitor cells [1].

Our studies clearly indicate that TAp73β is able to suppress the subpopulation of hepatocyte-like cells together with the promotion of cholangiocyte-like cells in the Hep3B and Huh7 cell lines. The suppression of hepatocyte lineage markers by TAp73β reported here reminds us of an earlier report demonstrating the direct repression of AFP transcription by both TAp73β and TAp73β via chromatin modification in HCC cells [49]. Our report confirms AFP repression by TAp73β, and associates this molecular event with hepatocellular cell fate change. Also, in a previous study, YAP activation has been shown to suppress hepatocyte differentiation through the suppression of the HNF4α in resident liver stem cells [50]. Taken together, we propose that TAp73β can play a promalignant role in HCC by inducing dedifferentiation of hepatocyte lineage cells toward cholangiocyte/stem cell-like phenotype through YAP activation. However, the blockade of stem/progenitor cell differentiation towards mature hepatocytes is also a plausible mechanism.

## 4. Materials and Methods

### 4.1. p73 Expression Analysis in Hepatocellular Carcinoma Using TCGA Data

Descriptive statistics of the p73 isoforms expression values were performed for non-tumor liver (normal; n = 50) and hepatocellular carcinoma (tumor; n = 371). The Shapiro-Wilk normality test was used to analyze whether the continuous variables were distributed normally. Since the expression values were not normally distributed, the Mann-Whitney U test was used to compare the median values of each group. IBM SPSS Version 25.0 statistical software and GraphPad Prism 7 (GraphPad Software, San Diego, CA, USA) were used for statistical analysis. For the survival analysis of the p73 gene, we utilized the “TCGAanalyze_survival” function of the TCGAbiolinks (v2.13.3) R package. Patients who had clinical information with at least 120 days of follow-up information or a death event were included in the study. Additionally, patients were divided into two groups, high (FPKM ≥ 0.5) and low (FPKM < 0.5) expression levels, based on fragments per kilobase of transcript per million (FPKM) value. The graphical visualizations of the results of statistical calculations were performed with the ggplot2 package (v3.2.1) of the R (v3.6.2) statistical computation environment. The significance level was taken as 0.05 in group comparisons for survival analysis.

### 4.2. Cell Culture

RPMI (Cat. 11875168, Thermo Fisher Scientific, Waltham, MA, USA) media was used for HCC cell lines, Huh-7, Hep3B, Hep3B-TR, HepG2, Hep40, Mahlavu, FOCUS, SNU398, SNU182, SNU423, SNU449, SNU475, SNU387. Complete medium was prepared by adding penicillin-streptomycin (100 U, 100 μg) (Cat. 15140148, Thermo Fisher), fetal bovine serum (FBS) (Cat. 16000036, Thermo Fisher Scientific) 50 mL, the non-essential amino acid mixture (Cat.10270106, Thermo Fisher Scientific) in 1× final concentration to the media. In routine cell culture procedures, cells were passaged when they reached a density of 70–80%.

### 4.3. RNA Interference

Hep3BTAp73β cells were transfected with equal amounts of ON-TARGETplus SMARTpool human p73 siRNA (Cat. L-003331-00-0010, GE Healthcare Dharmacon, Lafayette, CO, USA) or scramble siRNA by Lipofectamine RNAiMAX (Cat. 13778075, Invitrogen, Carlsbad, CA, USA) according to the manufacturer’s protocol. Proteins were harvested and analyzed for p73, HNF4α, ALB expression by western blot.

### 4.4. Microarray Analysis

Hep3BTAp73β cells were treated with doxycycline (1 μg/mL) for 12 h, 24 h and 48 h. Total RNA extractions were performed according to kit instructions (Cat. 740955.50, MN Macherey-Nagel, Düren, Germany) RNA quality was assessed using Agilent Bioanalyzer (Agilent, Santa Clara, CA, USA). Microarray analysis of RNA samples was performed using Affymetrix GeneChip Human Genome U133 Plus 2.0 arrays. The microarray data were collected and stored with Gene-Chip Operating Software (Affymetrix, Santa Clara, CA, USA). The quality of the arrays was evaluated at the image level by uploading the CEL files to the RMAExpress software (http://rmaexpress.bmbolstad.com). AffyPLM was used to assess the quality of the dataset. NUSE and RLE plots were drawn and outliers with high deviation from the average probe intensity value were excluded from further analyses. RMA normalization and class comparison analyses were performed using BRB-ArrayTools V4.5.0 [51]. The microarray data reported in this paper have been deposited in the Gene Expression Omnibus (GEO) database under accession numbers of GSE162860.

### 4.5. Gene Set Enrichment Analysis

GSEA was performed using microarray samples of 12 h, 24 h and 48 h Dox− and Dox+ Hep3BTAp73β cells, the hallmark gene set collection of the Molecular Signatures Database (MSigDB) V6.2 [33] and the GSEA V3.0 software of the Broad Institute [52].

### 4.6. Apoptosis Assay

Hep3BTAp73β cells were seeded into a 6-well cell culture plate. After 24 h, cells were treated with doxycycline (1 μg/mL) −/+ for 72 h. The media was removed, cells were washed twice with 1X PBS and treated with trypsin-EDTA (0.25%) (Thermo Fisher Scientific). Apoptosis assay was performed by following the kit instructions (Cat. 559763, Becton Dickinson, Franklin Lakes, NJ, USA) and analysis was performed by BD FACSCanto II (Becton Dickinson).

### 4.7. Sulforhodamine B (SRB) Assay

To analyse cell survival, 4 × 10^3^ cells/well were seeded into 96-well plates and incubated in a 37 °C incubator. After a 24 h, cells were treated with doxycycline (1 μg/mL) −/+ for 0 h, 24 h, 48 h and 72 h. Then, cells were fixed with 10% ice-cold trichloroacetic acid (TCA) (Cat. T6399, Sigma-Aldrich, St. Louis, MO, USA) and incubated in the dark at −20 °C for 20 min and TCA washed away. Then, the plates were stained with 0.4% Sulforhodamine B (SRB) (Cat. S1402, Sigma-Aldrich) in 1% acetic acid (CAS:64-19-7, Sigma-Aldrich) solution. Following staining, the plates were washed away using 1% acetic acid. The absorbance measured following the bound stain was solubilized using 10 mM Tris-Base (T1503, Sigma-Aldrich). The OD values were obtained at 512 nm using Varioskan, FLASH (Thermo Fisher Scientific).

### 4.8. Cell Cycle Analysis

To determine the effect of the ectopic expression of TAp73β on cell cycle progression, Hep3BTAp73β cells were cultured for 24 h, and they were treated with or without doxycline medium (1 μg/mL) for 72 h. After trypsinization cells were fixed in cold 70% ethanol for 10 min, then stained with propidium iodide (PI) solution (Cat. P4864,Thermo Fisher Scientific) 1 μg/mL PI and 0.125% RNaseA; (Cat. 12091021, Thermo Fisher Scientific) at 37 °C for 15 min. Approximately 1 × 10^4^ cells were analysed for per condition by BD FACSCanto II (Becton Dickinson). Then, the percentage distribution of cells in each phase was determined using Flowjo software.

### 4.9. Protein Analysis

The cells were lysed using RIPA lysis buffer containing 1 mM Na3VO2, 1 mM NaF, and 1X protease inhibitor cocktail (Cat. 11697498001, Roche, Basel, Switzerland). The antibodies used for protein immunodetection were described in Table S4. Anti-rabbit and anti-mouse horseradish peroxidase (HRP)-labelled IgGs (Cat. 7074; Cat. 7076, Cell Signaling Technology, Danvers, MA, USA) were used as secondary antibodies. Chemiluminescence signal was detected with ECL substrate (Pierce Biotechnology, Waltham, MA, USA) by using Chemi-Smart device (VILBER LOURMAT). Densitometric comparison of the western blots bands was performed using ImageJ (National Institutes of Health, Bethesda, MD, USA) software by normalized to the loading control (β-actin). All uncropped WB images were shown in Figure S3.

### 4.10. Immunofluorescence Assay

Hep3BTAp73β cells were seeded on a coverslip in the 12-well plate. On the next day, cells were treated with the complete media with or without doxycycline (1 μg/mL) for 72 h, then fixed with 4% paraformaldehyde in PBS for 5 min at RT. The cells were permeabilized with PBS containing 0.25% Triton-X-100 for 5 min. Then, the slides were incubated in 1X PBS-0.1% Tween-20 (PBS-T) containing 10% FBS for 1 h for blocking and incubated with primary antibodies (Table S4) for 12 h at +4 °C. Slides were washed four times with 1X PBS-T and incubated with secondary antibodies for 1 h at room temperature, and then slides were washed with 1XPBS-T five times. Finally, slides were mounted using a mounting medium and analyzed using confocal laser scanning microscope (Zeiss Oberkochen, Germany).

### 4.11. Three-Dimensional Cell Culture

Cells were suspended in 70% Matrigel (Cat. 356231, Corning, NY, USA), and 50 µL of Matrigel and cell suspension complex were seeded into a low adherent 24-well plate. Then cells were incubated in a 37 °C incubator containing 5% CO_2_ for 15 min, and 1 mL of complete media was added to each well. After 4 days, cells were treated with 1 µg/mL Doxycycline for 5 days. The medium was changed every 2 days. Then 3D cell structures were embedded into paraffin, and sections were taken to perform H&E and IHC staining [53].

### 4.12. Wound Repair Assay

Following Hep3BTAp73β cells were treated with doxycycline (1 μg/mL) −/+ medium for 72 h, wound healing assay was performed. Equal numbers of cells were re-plated in 6-well plate in triplicate and grown to confluence overnight in the doxycycline (1 μg/mL) −/+ medium. The next day monolayer was wounded using a sterile 200-μL pipette tip, the media was changed to remove debris, and imaged at 0 h and 24 h. The average percent of wound healing was determined based on 3 measurements of the wound area by using ImageJ (National Institutes of Health) software.

### 4.13. Zebrafish Metastasis Assay

Zebrafish was used as a host organism for xenograft studies as described previously [43,53]. Briefly, Hep3BTAp73β cells were treated with doxycycline (1 μg/mL) −/+ media for 72 h, and cells were detached from culture dishes using 0.25% Trypsin-EDTA, then washed twice with 1X PBS at room temperature. The cells were stained with 2 μg/mL Vybrant DiI (Cat.V22885, Thermo Fisher Scientific) diluted in PBS. Cells were washed once with FBS, twice with 1X PBS, then resuspend in 10% FBS in 1× PBS. Approximately 300 cells were injected into the yolk sac of the AB^+/+^ wild type 2dpf zebrafish embryos. Phenol red (Cat. P0290, Sigma-Aldrich) was used for the visibility of cell suspension during the injection. Doxycycline-treated and untreated cells were injected into two different embryo groups (n = 30 for each group). The next day, the larvae which contained cells in their yolk sac and not yet dispersed to other parts of their bodies were blindly selected. Then doxycycline (5 μg/mL) was added on water and treatment lasted for 3 days. Five or more cancer cells migrating from the injection site was accepted as metastasis and counted blindly under a stereomicroscope (Olympus, Tokyo, Japan). The experiment was repeated three times.

### 4.14. Reverse Transcriptase Polymerase Chain Reaction

Hep3BTAp73β cells were treated with doxycycline 1 μg/mL for 72 h then total RNA was exracted using the NucleoSpin RNA isolation kit (Cat. 740955.50, MN Macherey-Nagel) and 1 μg RNA was converted to cDNA using a Maxima First Strand cDNA Synthesis kit (Cat: 4309155, Thermo Fisher Scientific). Sybr green master mix (K1642, Thermo Fisher Scientific) was used for amplified of *p73*, *EpCAM*, *CYP3A4*, *AFP*, *HNF4* genes. Primer sequences for *EpCAM*, *CYP3A4*, *AFP*, *HNF4A* and *ALB* genes were described in a previous study [54]. The primer sequences for human *p73* is as follows: R-TCATCCACATCTGCGAGAGA, F-TTCAACGAAGGACAGTCTGC. The relative quantification was determined by using the the 2^−∆∆Ct^ method.

### 4.15. Ectopic Expression of TAp73 in HCC Cell Lines

To generate cells with an inducible ectopic expression of TAp73β, full length cDNA coding human TAp73β was cloned to pT-REx-DEST30 vector using Gateway cloning technology (Cat. 12301016, Thermo Fisher Scientific). Hep3B cells were co-transfected with the pcDNA6/TR vector (Cat. K10200 Thermo Fisher Scientific) and pT-REx-DEST30-TAp73β using Amaxa Nucleofector Technology (Lonza, Basel, Switzerland) according to the manufacturer’s instructions. Transfected cultures were selected with blasticidin (Cat. A1113903, Thermo Fisher Scientific) (4 μg/mL) and G418 (Cat. 10131035, Thermo Fisher Scientific) (1000 μg/mL) for three weeks. Resistant colonies were picked, expanded under selective conditions and tested for the inducibility of TAp73β expression in Hep3BTAp73β cells by western blot. Huh7 cells were transiently transfected with Empty vector and pT-REx-DEST30-TAp73β for 48 h using Fugene HD (Cat.E2311, Promega, Madison, WI, USA) then cell lysates were obtained to examine TAp73, ALB and HNF4α expressions at the protein levels by western blot. TAp73α, TAp73β and TAp73γ isoforms in pTRE expression vectors were ectopically expressed in Huh-7 to use as a control [55]. H1299 cells were transiently transfected with pcDNA3 expression plasmids encoding TAp73α and DNp73α isoforms were kindly provided by Dr. Karin Nylander.

### 4.16. Statistical Analysis

Statistical analyses were carried out using IBM SPSS Version 25.0 statistical software and GraphPad Prism 7. Statistical methods included Mann-Whitney U, Shapiro-Wilk normality, ANOVA and Student’s *t*-tests. Differences between groups were considered significant at * *p* < 0.05, ** *p* < 0.001, and *** *p* < 0.0001

## 5. Conclusions

This study clearly demonstrates that TAp73 isoforms are overexpressed in a large set of HCC tumors. At the cellular level TAp73β overexpression provoked major changes in HCC cells correlating with decreased proliferation as well as dedifferentiation from a hepatocyte-like phenotype towards cholangiocyte-like phenotype. These changes occurred as a result of landscape expression changes in sets involved in variety of cellular processes such as cell cycle, growth signaling, stress response, metabolism and development. This role of *p73* gene needs to further explored in order to development new approaches for clinical evolution and therapy of HCC.

## Data Availability

The data presented in this study are openly available in the Gene Expression Omnibus (GEO) database under accession numbers of GSE162860.

## Supplementary Materials

The following are available online at https://www.mdpi.com/2072-6694/13/4/783/s1, Figure S1: The effect of TAp73β on apoptosis in Hep3B cells. Figure S2: The effect of TAp73β on CK18, B-catenin, HepPar-1 and Arginase expression. Figure S3: uncropped WB images. Table S1: The list of at least two-fold differentially expressed genes between Dox− and Dox+ Hep3BTAp73β cells in any time points. Table S2: TAp73β -regulated hallmark gene sets. Table S3: TAp73β -affected Hallmark Gene Sets. Table S4: Antibodies used for Immunofluorescence, Western Blot and Immunohistochemistry.
