# Supplementary material for: TAp73β Can Promote Hepatocellular Carcinoma Dedifferentiation
Source: Cancers (Basel). 2021 Feb 13;13(4):783. doi: 10.3390/cancers13040783 (PMC7918882; doi:10.3390/cancers13040783)
Supplement: Supplementary file 1 [file cancers-13-00783-s001.zip › cancers-1017984-proofreading suppl/Sup_material.docx]

**Supplemental Materials and Methods**

**
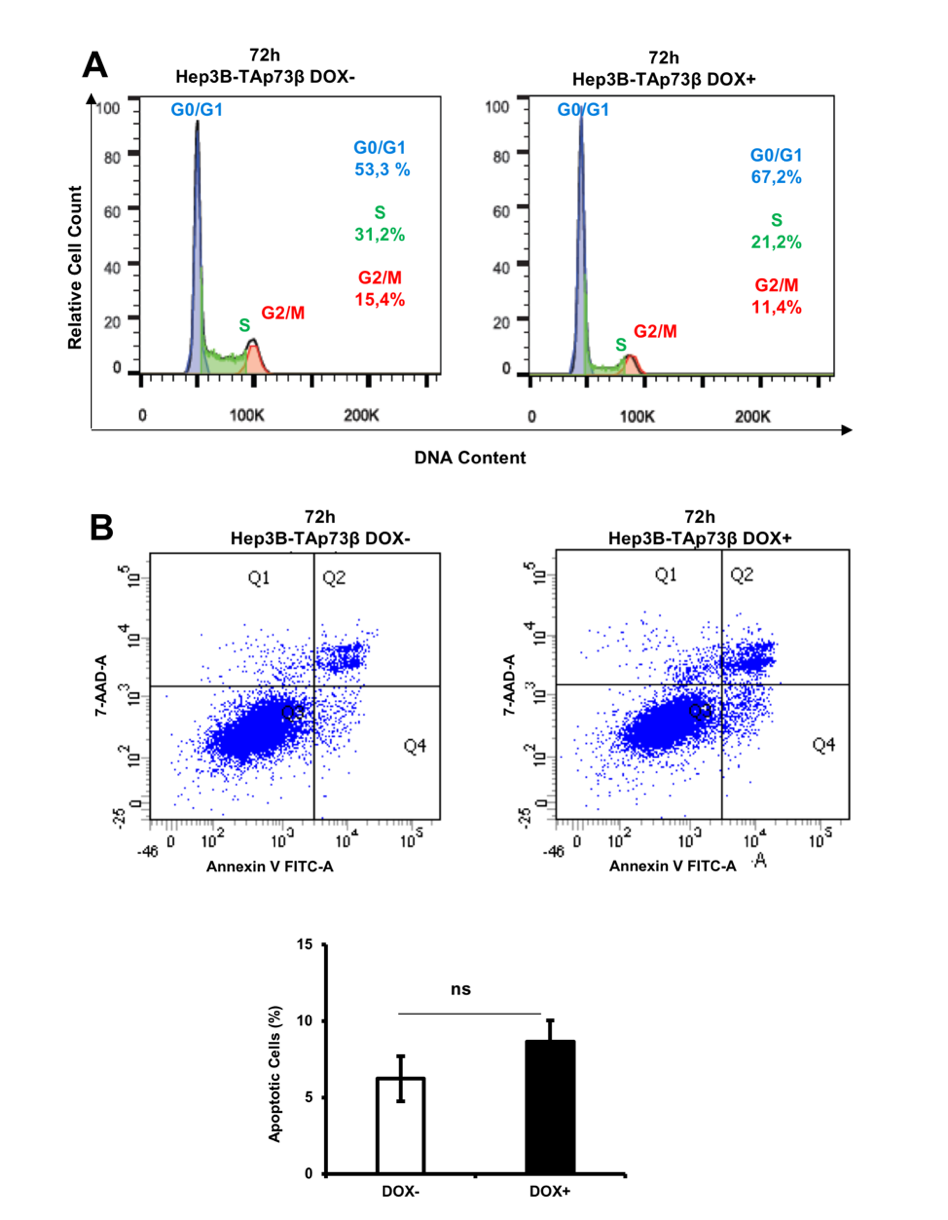
**

**Figure S1. The effect of TAp73**β **on apoptosis in Hep3B cells.** Hep3BTAp73 cells were grown in the absence (DOX-) or the presence (Dox+) of Doxycycline (1 µg/ml) for 72 hours. **(A)** Cell cycle distribution as determined using flow cytometric DNA content analysis. Cell population in sub-G1, G1, S and G2/M was determined using FlowJo software. **(B)** Cells were stained with Annexin V/7-ADD and analyzed by flow cytometry. ns: not significant.

**
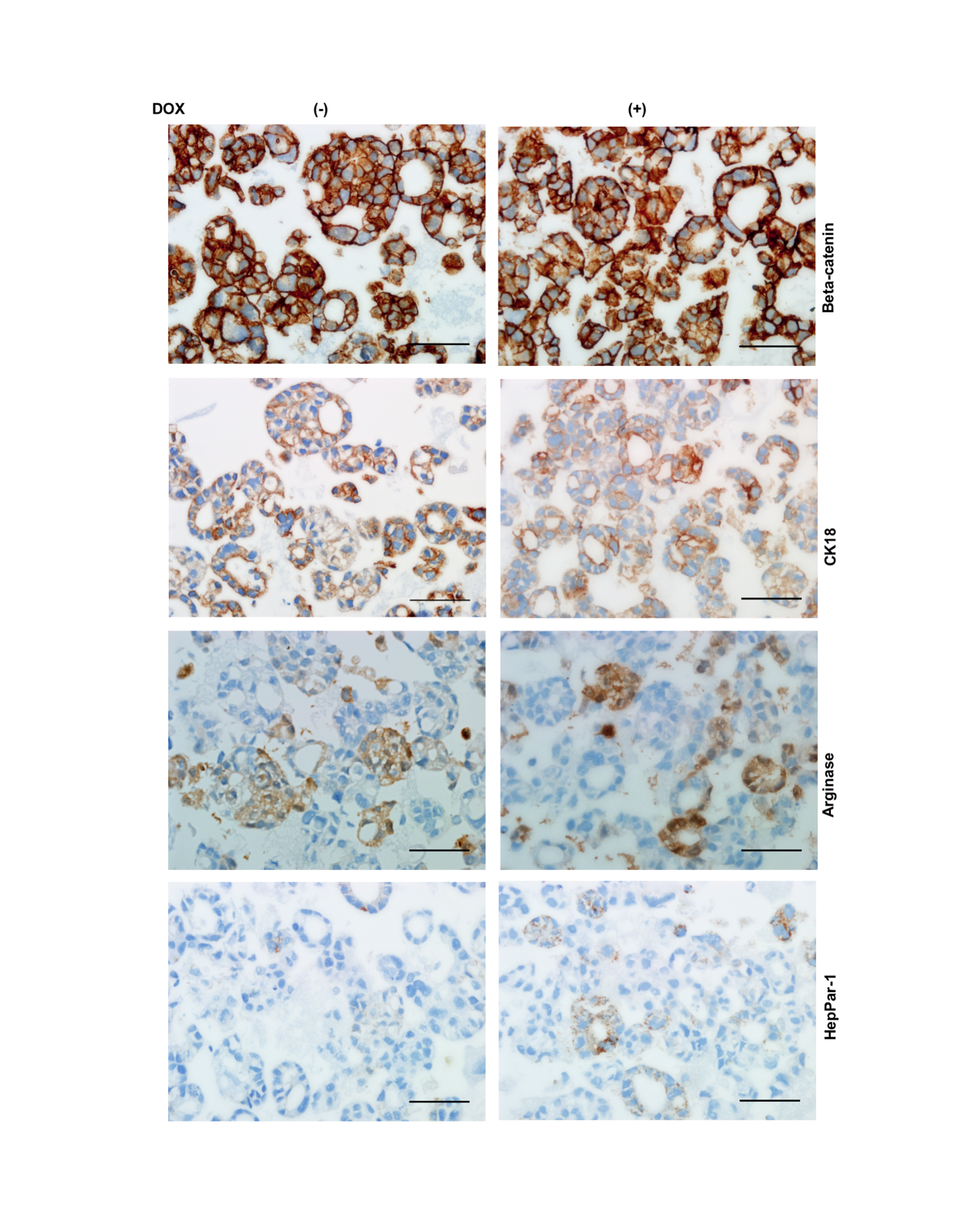
**

**Figure S2. The effect of TAp73**β **on CK18, B-catenin, HepPar-1 and Arginase expression.** IHC staining for CK18, β-catenin, HepPar-1 and Arginase was performed using formaldehyde-fixed paraffin-embedded organoids that have been grown for five days in the presence (DOX+) or absence (DOX-) of Doxycycline. Scale bar=100μM

**Supplemental Tables**

**Table 3.** TAp73-affected Hallmark Gene Sets

| **TAP73 EFFECT** | **CELLULAR PROCESSES** | **GENE SETS** |
| --- | --- | --- |
| Induction | Stress response | Genes involved in p53 pathways and networks |
|  |  | Genes up-regulated in response to hypoxia |
|  |  | Genes up-regulated by reactive oxygen species |
|  |  | Genes down-regulated in response to ultraviolet radiation |
|  |  | Genes defining inflammatory response |
|  |  | Genes encoding components of peroxisome |
|  |  | Genes encoding components of blood coagulation system |
| Induction | Growth signaling | Genes defining early and late estrogen response |
|  |  | Genes up-regulated by KRAS activation |
|  |  | Genes up-regulated through activation of mTORC1 |
| Repression | Growth signaling | Genes regulated by MYC |
| Repression | Cell cycle | Genes encoding cell cycle related targets of E2F transcription factors. |
|  |  | Genes involved in the G2/M checkpoint, |
|  |  | Genes important for mitotic spindle assembly. |
| Induction | Development | Genes upregulated during adipocyte differentiation |
|  |  | Genes encoding components of apical junction complex |
|  |  | Genes Defining Epithelial-Mesenchymal Transition |
|  |  | Genes involved in development of skeletal muscle |
|  |  | Genes upregulated by Hedhehog signaling |
|  |  | Genes up-regulated during formation of blood vessels |
| Induction | Immune response | Genes up-regulated during transplant rejection |
|  |  | Genes up-regulated by STAT5 in response to IL2 stimulation. |
|  |  | Genes up-regulated in response to Intereferon alpha |
|  |  | Genes up-regulated in response to Interferon gamma |
|  |  | Genes regulated by NF-kB in response to TNFA |
| Induction | Metabolism | Genes involved in metabolism of bile acids and salts |
|  |  | Genes encoding proteins involved in glycolysis and gluconeogenesis. |
|  |  | Genes encoding proteins involved in processing of drugs and other xenobiotics. |

**Table 4.** Antibodies used for Immunofluorescence, Western Blot and Immunohistochemistry

| **Antibody Name** | **Catalog Number or Reference** | **Dilution for WB** | **Dilution for IF** | **Dilution for IHC** |
| --- | --- | --- | --- | --- |
| FLp73 | Sayan et. al., 2005 | 1/2000 | 1/200 | - |
| IHC00197 | Bethyl Lab. (A300-126A) | 1/500 | - | - |
| ΔNp73-1.1 | Veselska et al., 2013; Nekulova et al., 2013 | 1/1000 |  |  |
| Albumin | Santa-Cruz Biotech (sc30121) | 1/1000 | - | - |
| ZO-1 | Invitrogen (40-2200) | 1/1000 | - | - |
| EpCAM | Sigma (E6011) | 1/1000 | - | - |
| CFTR | Santa-Cruz Biotech (sc10747) | 1/1000 | - | - |
| HNF4A | Santa-Cruz Biotech (sc8987) | 1/1000 | - | - |
| YAP/TAZ | Cell Signaling (D24E4) | 1/1000 | 1/100 | - |
| HNF-1B | Santa-Cruz Biotech (sc130407) | 1/1000 | - | - |
| LGR5 | Novus (Nbp-1-40567) | 1/1000 | - | - |
| Bax | Santa-Cruz (sc20067) | 1/1000 | - | - |
| JAG1 | Santa-Cruz Biotech (sc6011) | 1/1000 | - | - |
| NOTCH1 | Santa-Cruz Biotech (sc6014R) | 1/1000 | - | - |
| CK19 | **Cell Marque (A53-B/A2.26)** | - | - | 1/100 |
| AFP | **Cell Marque (203A-17)** | - | - | 1/100 |
| CK18 | **Cell Marque (**EP17/EP30) | - | - | 1/100 |
| Arginase-1 | **Cell Marque (SP156)** | - | - | 1/100 |
| HepPAR-1 | **Cell Marque (EP265)** | - | - | 1/100 |
| B-catenin | **Cell Marque (**AC-0034) | - | - | 1/100 |
| B-actin | Abcam (ab8226) | 1/1000 | - | - |
