# Supplementary material for: TAp73β Can Promote Hepatocellular Carcinoma Dedifferentiation
Source: Cancers (Basel). 2021 Feb 13;13(4):783. doi: 10.3390/cancers13040783 (PMC7918882; doi:10.3390/cancers13040783)
Supplement: Supplementary file 1 [file cancers-13-00783-s001.zip › cancers-1017984-proofreading suppl/Figure S3 WB data.pdf]

Figure 1D

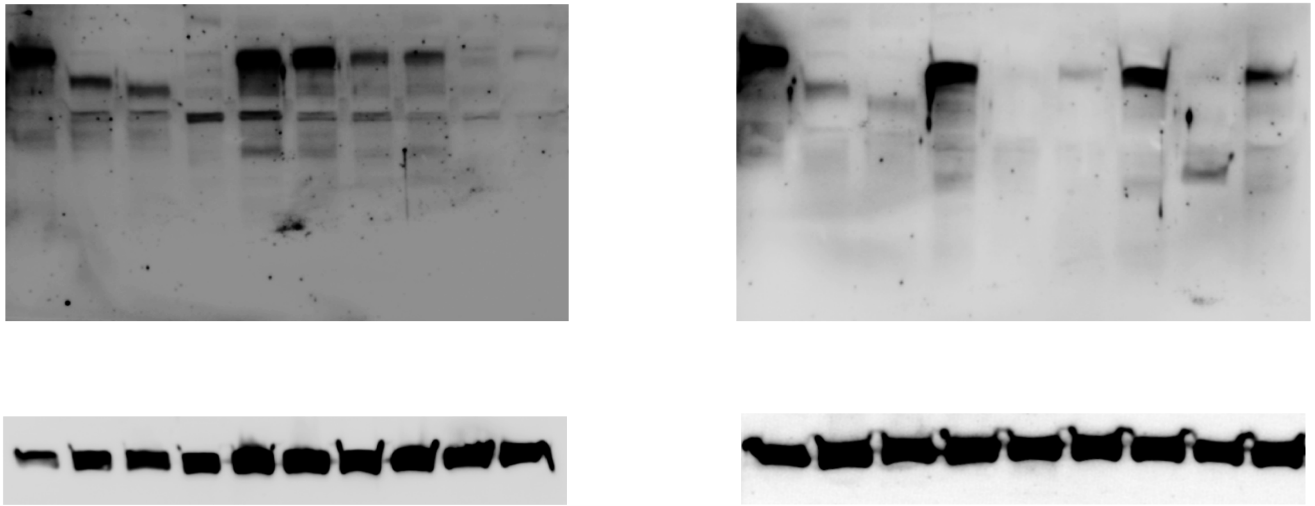

Antibody=ab: FLp73

Antibody=ab: Beta-actin

Figure 2

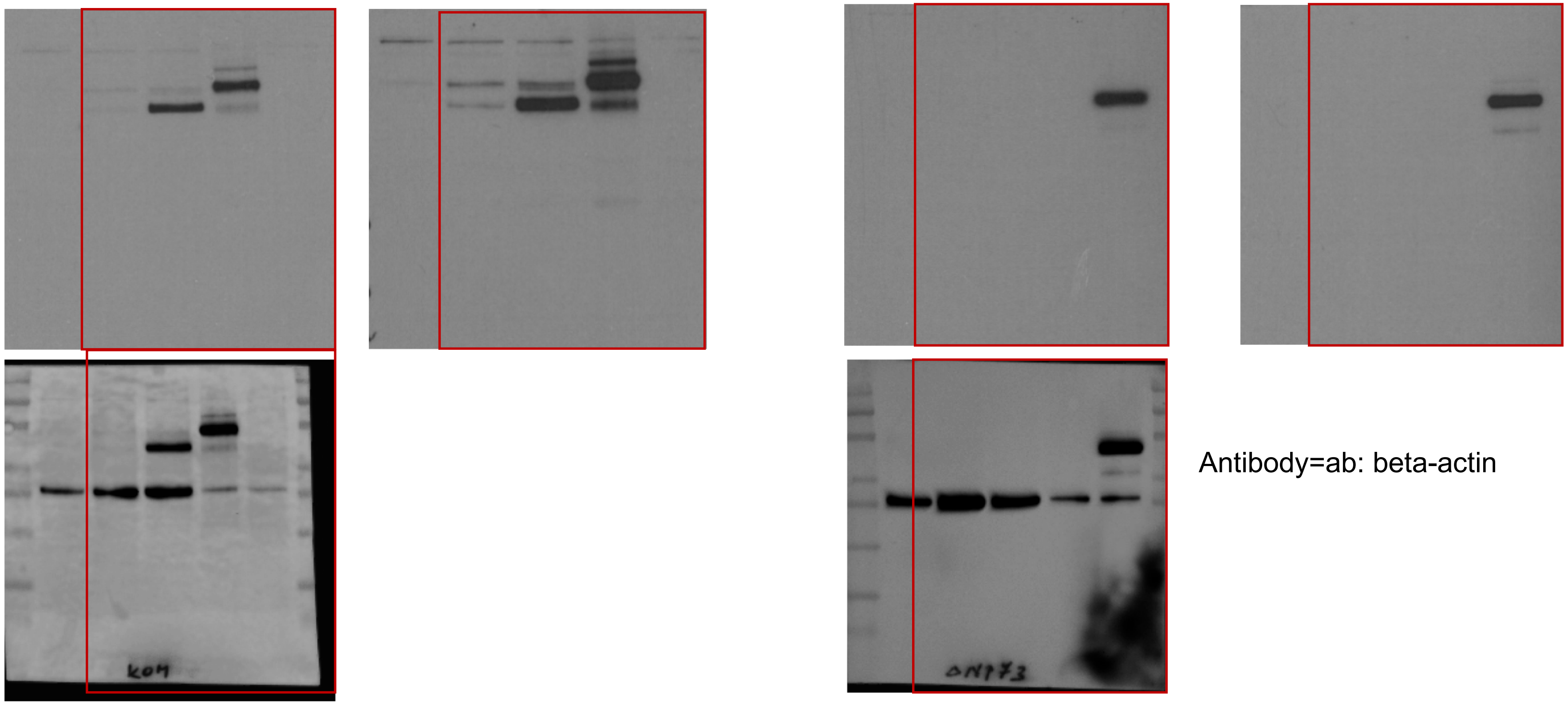

Antibody=ab: IHC00197 (Anti-TAp73)

Antibody=ab: ΔNp73-1.1

Antibody=ab: beta-actin

Antibody=ab: beta-actin

Fig. 4A

Antibody=ab: FLp73

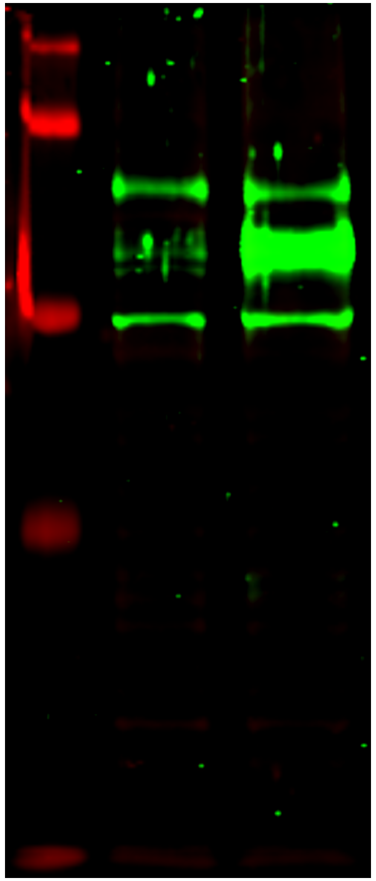

Antibody=ab: Beta-actin

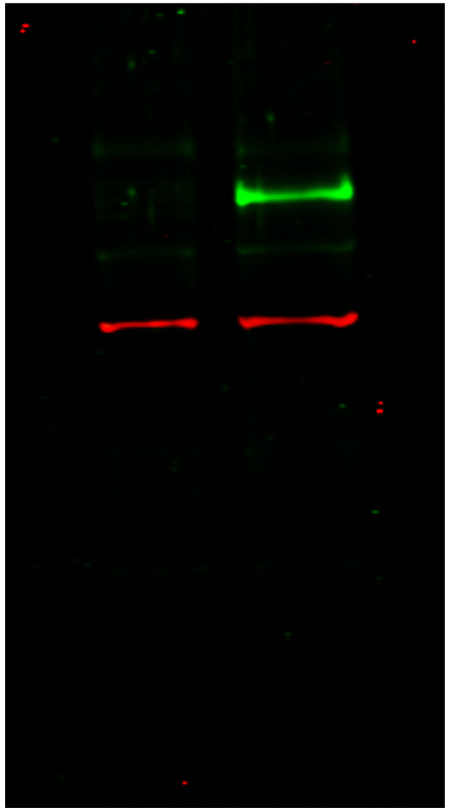

Fig. 4A (continued)

Antibody=ab: Bax

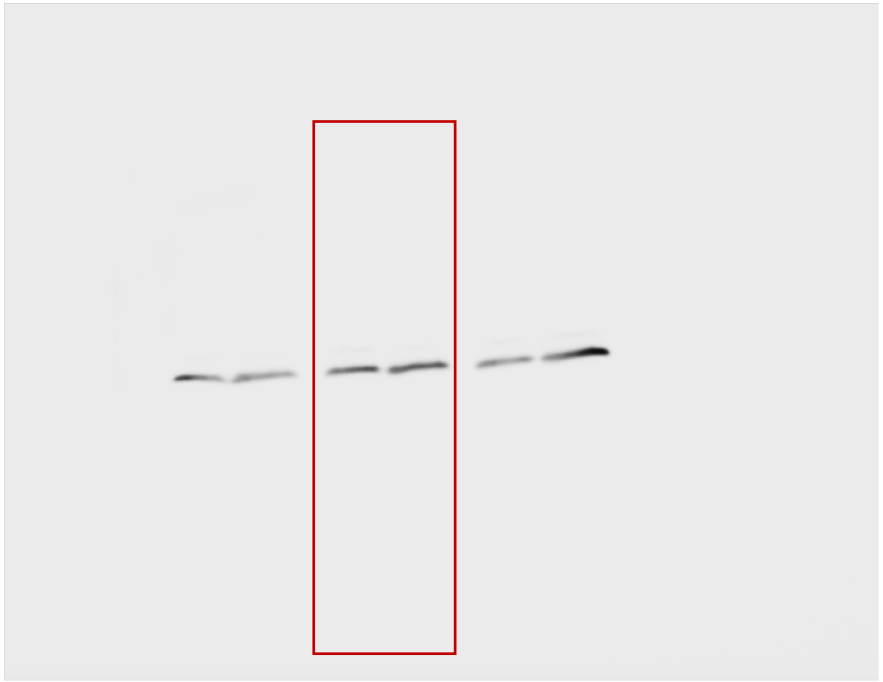

Antibody=ab: Beta-actin

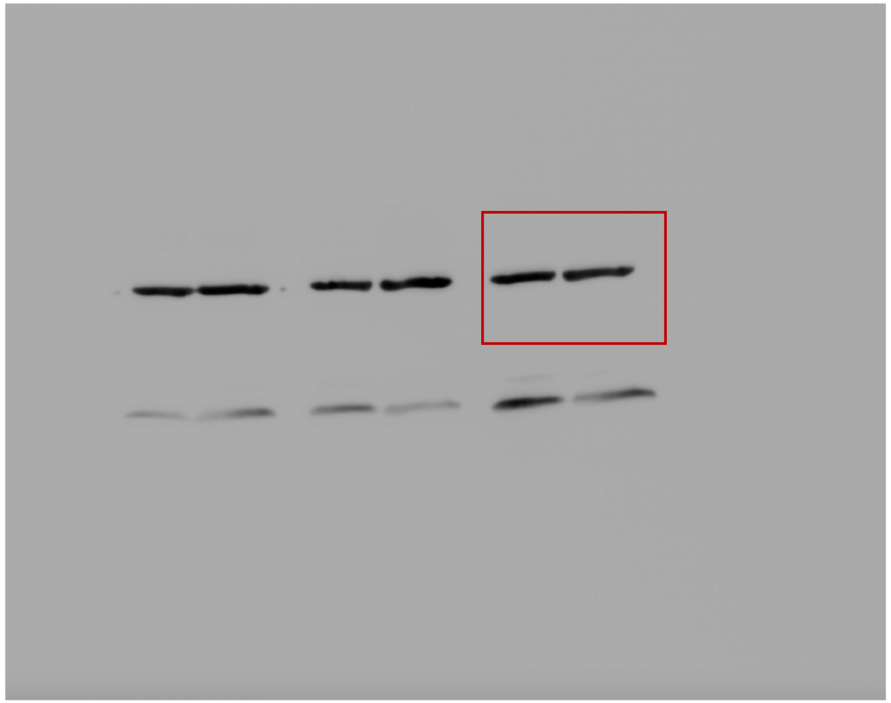

Fig. 4A (contiuned)

Antibody=ab: YAP/TAZ

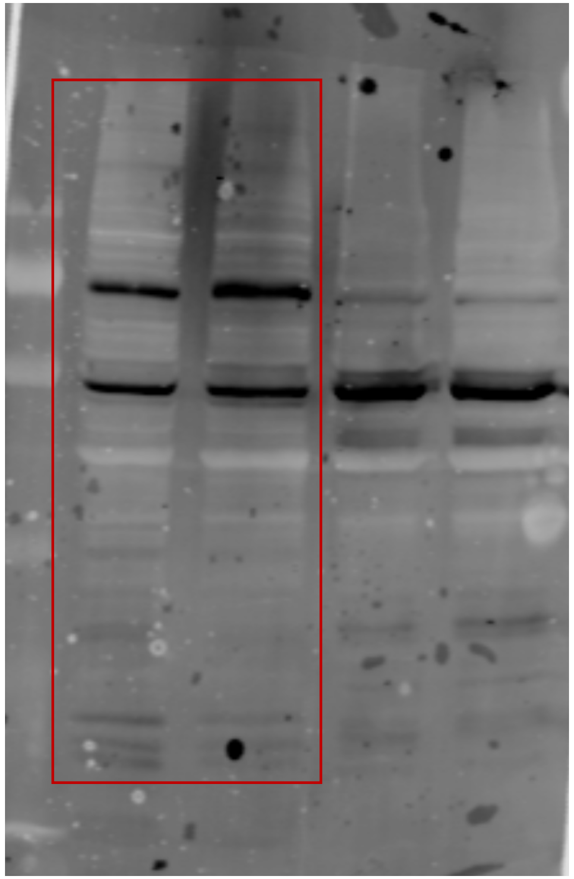

Antibody=ab: Beta-actin

Antibody=ab: Calnexin

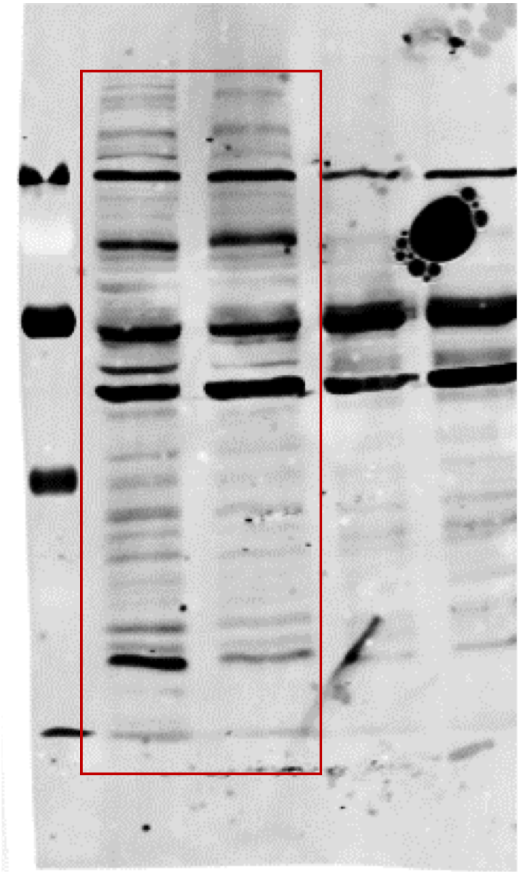

Fig.5A

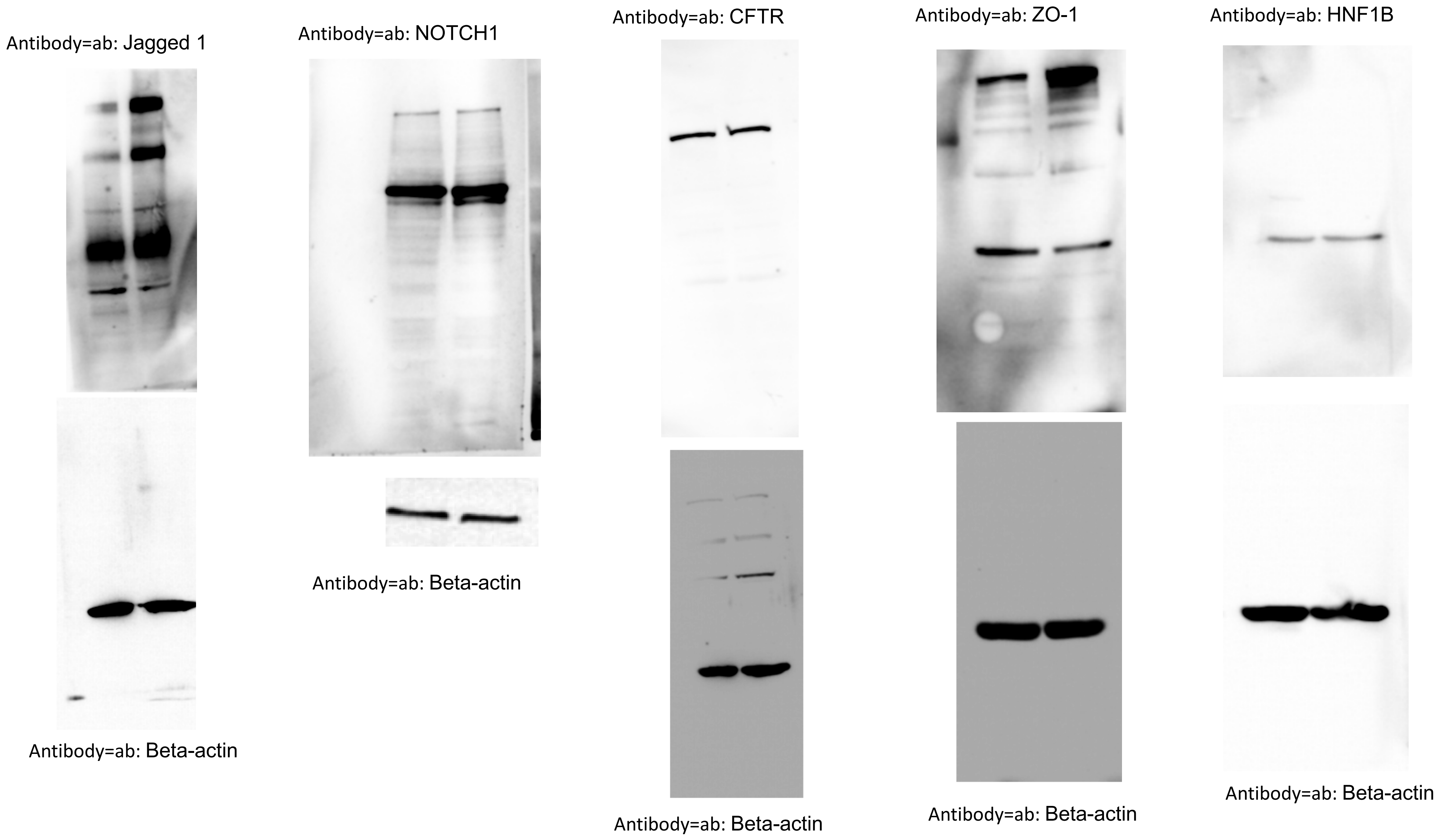

Antibody=ab: Jagged 1
Antibody=ab: Beta-actin
Antibody=ab: NOTCH1
Antibody=ab: Beta-actin
Antibody=ab: CFTR
Antibody=ab: Beta-actin
Antibody=ab: ZO-1
Antibody=ab: Beta-actin
Antibody=ab: HNF1B
Antibody=ab: Beta-actin

Fig.5C

Antibody=ab: EpCAM

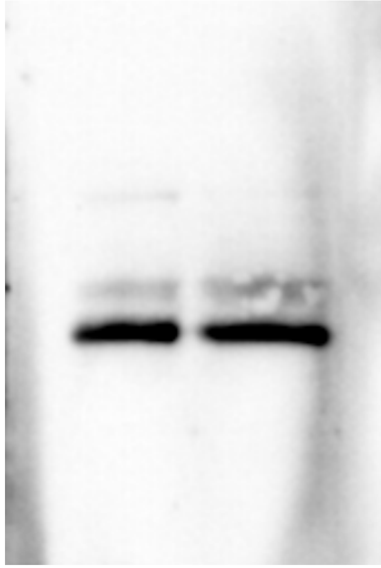

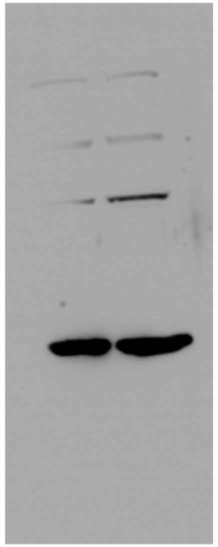

Antibody=ab: Beta-actin

Antibody=ab: LGR5

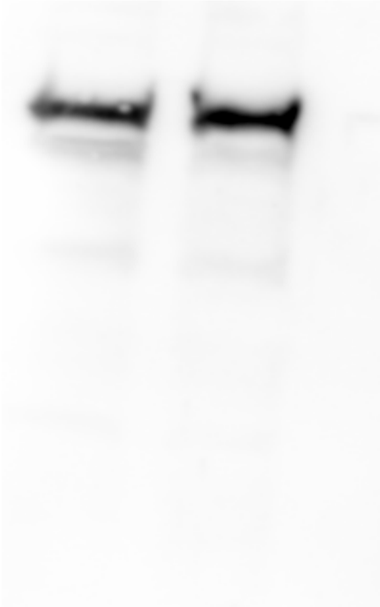

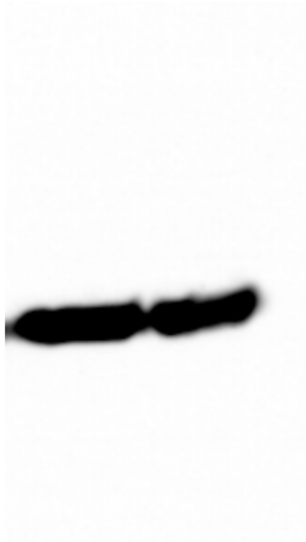

Antibody=ab: Beta-actin

Fig.5D

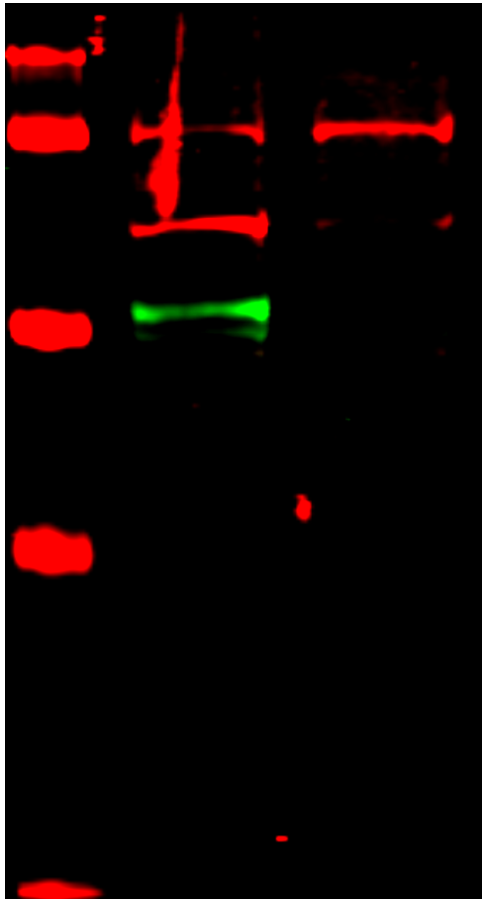

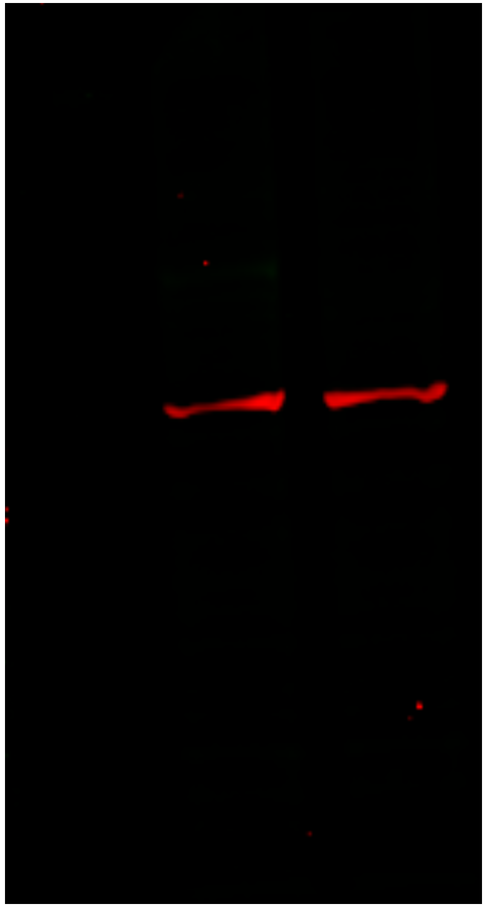

Figure 5 E

Antibody=ab: FLp73 Antibody=ab: HNF4A Antibody=ab: ALB

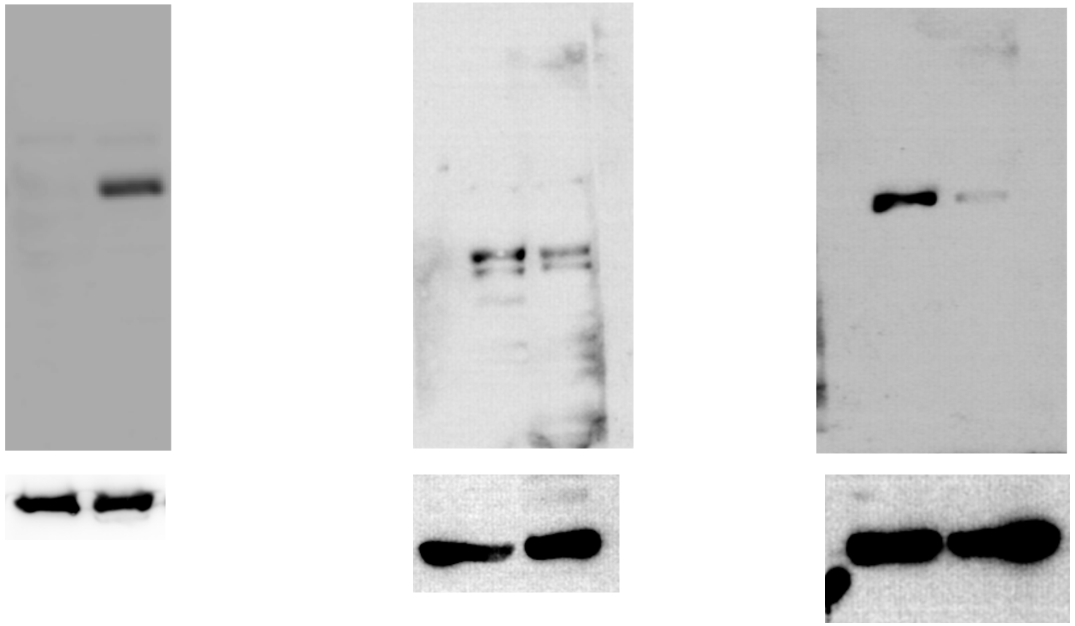

Antibody=ab: Beta-actin Antibody=ab: Beta-actin Antibody=ab: Beta-actin

Figure 5F

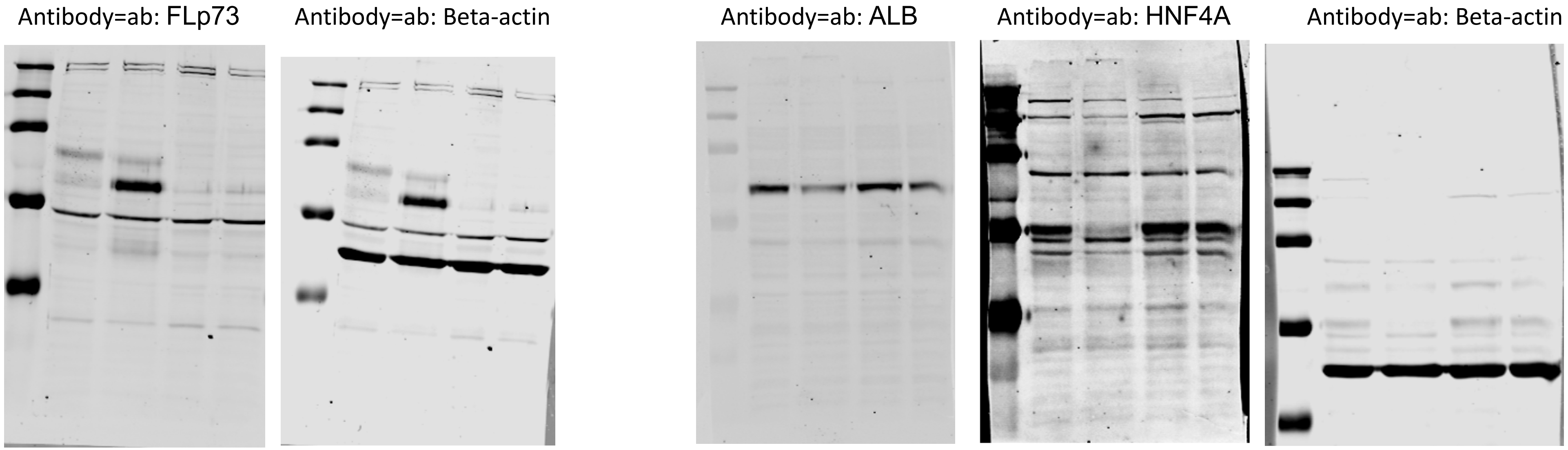
Antibody=ab: FLp73
Antibody=ab: Beta-actin
Antibody=ab: ALB
Antibody=ab: HNF4A
Antibody=ab: Beta-actin
